# Bruton's tyrosine kinase inhibitor BMS-986142 in experimental models of rheumatoid arthritis enhances efficacy of agents representing clinical standard-of-care

**DOI:** 10.1371/journal.pone.0181782

**Published:** 2017-07-24

**Authors:** Kathleen M. Gillooly, Claudine Pulicicchio, Mark A. Pattoli, Lihong Cheng, Stacey Skala, Elizabeth M. Heimrich, Kim W. McIntyre, Tracy L. Taylor, Daniel W. Kukral, Shailesh Dudhgaonkar, Jignesh Nagar, Dana Banas, Scott H. Watterson, Joseph A. Tino, Aberra Fura, James R. Burke

**Affiliations:** 1 Immunosciences Discovery Biology, Bristol-Myers Squibb Research & Development, Princeton, New Jersey, United States of America; 2 Exploratory Clinical and Translational Research, Imaging, Bristol-Myers Squibb Research & Development, Princeton, New Jersey, United States of America; 3 Disease Sciences and Technology, Biocon Bristol-Myers Squibb Research Center, Syngene International Limited, Bangalore, India; 4 Discovery Translational Sciences, Bristol-Myers Squibb Research & Development, Princeton, New Jersey, United States of America; 5 Immunosciences Discovery Chemistry, Bristol-Myers Squibb Research & Development, Princeton, New Jersey, United States of America; 6 Department of Pharmaceutical Candidate Optimization, Bristol-Myers Squibb Research & Development, Princeton, New Jersey, United States of America; Charles P. Darby Children's Research Institute, 173 Ashley Avenue, Charleston, SC 29425, USA, UNITED STATES

## Abstract

Bruton’s tyrosine kinase (BTK) regulates critical signal transduction pathways involved in the pathobiology of rheumatoid arthritis (RA) and other autoimmune disorders. BMS-986142 is a potent and highly selective reversible small molecule inhibitor of BTK currently being investigated in clinical trials for the treatment of both RA and primary Sjögren’s syndrome. In the present report, we detail the in vitro and in vivo pharmacology of BMS-986142 and show this agent provides potent and selective inhibition of BTK (IC50 = 0.5 nM), blocks antigen receptor-dependent signaling and functional endpoints (cytokine production, co-stimulatory molecule expression, and proliferation) in human B cells (IC50 ≤ 5 nM), inhibits Fcγ receptor-dependent cytokine production from peripheral blood mononuclear cells, and blocks RANK-L-induced osteoclastogenesis. Through the benefits of impacting these important drivers of autoimmunity, BMS-986142 demonstrated robust efficacy in murine models of rheumatoid arthritis (RA), including collagen-induced arthritis (CIA) and collagen antibody-induced arthritis (CAIA). In both models, robust efficacy was observed without continuous, complete inhibition of BTK. When a suboptimal dose of BMS-986142 was combined with other agents representing the current standard of care for RA (e.g., methotrexate, the TNFα antagonist etanercept, or the murine form of CTLA4-Ig) in the CIA model, improved efficacy compared to either agent alone was observed. The results suggest BMS-986142 represents a potential therapeutic for clinical investigation in RA, as monotherapy or co-administered with agents with complementary mechanisms of action.

## Introduction

Despite availability of various disease-modifying anti-rheumatic drugs (DMARDs) for the management of rheumatoid arthritis (RA), less than a fifth of DMARD-experienced patients receiving anti-tumor necrosis factor (TNF) biologic treatment achieve a 70% improvement in disease activity (American College of Rheumatology [ACR]70 response), even when co-administered with methotrexate (MTX) [1]. New approaches to treatment are required, and increasingly the clinical community is looking toward combining treatments with new and complementary mechanisms of action to improve outcomes for patients with RA [2, 3].

B cells play multiple roles in RA pathobiology, including being a source of autoantibodies and inflammatory cytokines, and as antigen-presenting cells [4]. Bruton’s tyrosine kinase (BTK) is a key intracellular enzyme predominantly expressed in hematopoietic cells, including B cells, where it plays an essential role in B-cell receptor (BCR)-mediated activation, proliferation, cytokine production, and co-stimulatory molecule expression [5–8]. BTK is also expressed in myeloid cells [9], such as the monocytes, macrophages, neutrophils, and mast cells that infiltrate into the synovium in RA [10].

Immune complexes (ICs) containing immunoglobulin (Ig) G also play a critical role in the immunopathology of many autoimmune disorders. In RA, ICs are present in the joints and act on synovial macrophages to drive the production of cytokines, chemokines, and matrix metalloproteinases (MMPs) that are integral components of disease pathology. Indeed, Fcγ receptor IIa (FcγRIIa; cluster of differentiation [CD]32a) and FcγRIIIa (CD16) expression is increased in monocytes/macrophages from patients with RA, and these cells have been shown to produce higher levels of TNF-α and MMPs than those from healthy controls [11]. BTK has been shown to be essential in both the signal transduction pathway downstream of these activating IgG IC receptors as well as the subsequent expression of pro-inflammatory cytokines and integrins [5, 12].

Beyond its critical role in B-cell function and inflammatory cytokine production, BTK directly regulates bone resorption in RA. Signal transduction through receptor activator of nuclear factor kappa-B (RANK), the receptor for RANK ligand (RANK-L) that drives osteoclast differentiation and activation, and mediates bone destruction in RA, has been reported to be regulated by BTK [13, 14].

Because of the important role of BTK in regulating key pathogenic pathways, BTK inhibitors are under investigation as treatment options for various autoimmune diseases, including RA [5, 12, 15–19]. The selective inhibition of B-cell activation provided by a BTK inhibitor provides a novel opportunity to treat disease and potential for combination with complementary mechanisms.

Our efforts have identified BMS-986142 as a novel, small-molecule, oral, reversible inhibitor of BTK. Herein, we describe results of *in vitro* and *in vivo* studies conducted to characterize the pharmacology of BMS-986142 as a potential therapy in RA. Importantly, we characterize the effects of BMS-986142 in mouse models of RA, as monotherapy and in combination with the current standards of care.

## Materials and methods

### Synthesis of BMS-986142

BMS-986142 (6-fluoro-5-(R)-(3-(S)-(8-fluoro-1-methyl-2,4-dioxo-1,2-dihydroquinazolin-3(4H)-yl)-2-methylphenyl)-2-(S)-(2-hydroxypropan-2-yl)-2,3,4,9-tetrahydro-1H-carbazole-8-carboxamide) was synthesized as described previously [20].

### *In vitro* studies

#### Primary kinase assays

Human recombinant BTK (0.6 nM, His-tagged; Invitrogen™, Grand Island, NY, USA) was incubated at room temperature with 1.5 μM fluoresceinated peptide substrate (FITC-AHA-GEEPLYWSFPAKKK-NH2), 20 μM adenosine triphosphate (ATP), and BMS-986142 in assay buffer (20 mM HEPES, 10 mM MgCl_2_, 0.015% Briji-35, and 4 mM dithiothreitol). Product turnover was quantitated using a Caliper LabChip 3000 (Caliper Life Sciences, Hopkinton, MA, USA). Similar assays were used for other kinases (Tec kinase, interleukin [IL]-2-inducible T-cell kinase, B-lymphocyte kinase, T cell-expressed kinase, and bone marrow-expressed kinase), with ATP concentrations equal to the apparent Michaelis constant (K_M_^app^) for each kinase. Interaction of BMS-986142 at 1000 nM with 384 protein and lipid kinases was examined by screening using competition binding assays at DiscoveRx (Fremont, CA, USA; formerly Ambit Biosciences) [21]. Interaction of BMS-986142 at 200 nM with a panel of 337 kinases was also examined by using enzymatic assays at Reaction Biology (Malvern, PA, USA). [^33^P]-ATP was used as a substrate at a concentration equal to K_M_^app^, and incorporation of radiolabel into peptide or protein substrates was measured.

#### Functional activity

Human peripheral blood and isolated B cells and monocytes were obtained from normal healthy volunteers at Bristol-Myers Squibb with approval of Bristol-Myers Squibb Environmental Health & Safety and with written informed consent from the donors. Human samples were collected from 2013 through 2016.

Stimulated expression of CD86 and CD69 on human peripheral blood B cells: The E-negative fraction of human peripheral blood mononuclear cells, isolated after removal of T lymphocytes by rosetting with sheep red blood cells, were stimulated in media containing 10% fetal bovine serum (FBS) and various concentrations of BMS-986142 for 18 hours at 37°C with AffiniPure F(ab’)2 fragment goat anti-human IgG + IgM (Jackson ImmunoResearch, West Grove, PA, USA) for determination of CD86 and CD69 expression. The cells were stained with fluorescein isothiocyanate (FITC)-conjugated mouse anti-human CD20 antibody (BD Pharmingen, San Diego, CA, USA) and either allophycocyanin (APC)-conjugated mouse anti-human CD86 monoclonal antibody (BD Pharmingen) or phycoerythrin (PE)-conjugated mouse anti-human CD69 monoclonal antibody (BD Pharmingen). The amount of CD86 or CD69 expression was quantitated by median fluorescence intensity (MFI) after gating on the CD20-positive B-cell population using fluorescence-activated cell sorting (FACS) analysis. For analogous experiments measuring the effect of CD40 or Toll-like receptor 4 (TLR4) stimulation on these endpoints, either human IZ-CD40L or lipopolysaccharide (LPS) was used to stimulate the cells. To determine the effect of BMS-986142 on CD86 expression in memory B cells, an additional marker was employed (PE-conjugated mouse anti-human CD27; BD Pharmingen) to identify CD27+CD20+ memory B cells.

For whole blood assays of BCR-stimulated CD69 expression on B cells, human whole blood with anticoagulant citrate dextrose solution A as anticoagulant was added with various concentrations of BMS-986142 and stimulated with 30 ***μ***g/mL AffiniPure F(ab’)2 fragment goat anti-human IgM (Jackson ImmunoResearch) and 10 ng/mL human IL-4 (Peprotech, Rocky Hill, NJ, USA) for 18 hours at 37°C with agitation. The cells were stained with FITC-conjugated mouse anti-human CD20 (BD Pharmingen) and PE-conjugated mouse anti-human CD69 monoclonal antibody (BD Pharmingen), lysed and fixed, then washed. The amount of CD69 expression was quantitated by MFI after gating on the CD20-positive B-cell population as measured by FACS analysis.

For whole blood assays of FcεRI (anti-IgE)-stimulated CD63 expression on basophils, human whole blood (EDTA-treated) was diluted fourfold and pre-incubated with various concentrations of BMS-986142 for 30 minutes at 37°C and then stimulated with 0.9 ***μ***g/mL goat anti-human IgE (Invitrogen) and stained with PE-conjugated mouse anti-human C-C chemokine receptor type 3 (CCR3) and FITC-conjugated mouse anti-human CD63 monoclonal antibody (American Laboratory Products Company [ALPCO], Salem, NH, USA) for 25 minutes at 37°C. The blood was lysed and fixed, then washed. The percentage of CD63+ cells was measured after gating on the CCR3-positive basophil population by FACS analysis.

BCR-stimulated IL-6 and TNF-α from human tonsillar B cells: Human tonsils were obtained through the National Cancer Institute-supported Cooperative Human Tissue Network, and tonsillar B cells were isolated as described above and suspended in media containing 10% FBS and various concentrations of BMS-986142. After 1 hour of incubation at 37°C, the cells were stimulated with 40 μg/mL AffiniPure F(ab’)2 fragment goat anti-human IgM (Jackson ImmunoResearch) and 10 ng/mL IL-4 (Peprotech). After an additional 4.5 hours of incubation, the supernatants were collected and the levels of IL-6 and TNF-α were measured using an enzyme immunoassay.

BCR-stimulated human tonsillar B-cell proliferation: Human B cells from tonsils, isolated in media containing 10% FBS and various concentrations of BMS-986142, were stimulated with AffiniPure F(ab’)2 fragment goat anti-human IgG + IgM (Jackson ImmunoResearch) and incubated at 37°C for 72 hours [22]. The cells were then labeled with [^3^H]-thymidine and incubated overnight at 37°C. Cells were then harvested onto filter plates and the amount of [^3^H]-thymidine incorporation, as a measure of B-cell proliferation, was determined using liquid scintillation counting.

BCR-stimulated calcium flux in Ramos B cells: Human Ramos (RA1) B cells, a Burkitt’s lymphoma cell line (ATCC, CRL-1596), pre-loaded with calcium indicator dye (BD Biosciences, San Jose, CA, USA) in media containing 10% FBS, were plated on black clear-bottom assay plates at a density of 1 × 10^6^ cells/mL (150,000 cells per well). BMS-986142 was added at various concentrations, and the cells were incubated for 1 hour at room temperature in the dark after which the plates were centrifuged and the cells, stimulated with rabbit anti-human IgM (Jackson ImmunoResearch) to induce a calcium flux, measured using a fluorometric imaging plate reader.

B-cell receptor (BCR)-stimulated phospholipase C (PLC)-γ2 phosphorylation in Ramos B cells: After 1 hour of pre-incubation of Ramos B cells in media containing 10% fetal bovine serum (FBS) with varying concentrations of BMS-986142 at 37°C, the cells were stimulated with AffiniPure F(ab’)2 fragment goat anti-human immunoglobulin (Ig)M (Jackson ImmunoResearch, West Grove, PA, USA) at 50 μg/mL for exactly 2 minutes at 37°C, followed by addition of ice-cold phosphate-buffered saline for quenching. The cells were pelleted and lysed, and PLCγ2 levels were measured by immunoblot using rabbit anti-human phosphoY759-PLCγ2 (Cell Signaling Technology, Danvers, MA, USA) and analyzed using the Odyssey Infrared Imaging System (Li-Cor Biosciences, Lincoln, NE, USA) with normalization to an actin control to ensure consistent loading.

BCR-stimulated Bruton’s tyrosine kinase (BTK) phosphorylation in Ramos B cells: After pre-incubation of Ramos B cells in media containing 10% FBS with varying concentrations of BMS-986142 at 37°C, the cells were stimulated with AffiniPure F(ab’)2 fragment goat anti-human IgM (Jackson ImmunoResearch) at 50 μg/mL for exactly 2 minutes at 37°C. Cells were then fixed and stained with an Alexa647-conjugated anti-phospho-BTK antibody, which specifically recognizes phosphorylated-Y551 (BD Biosciences, San Jose, CA, USA), and the amount of phospho-Y551 was quantitated by median fluorescence intensity (MFI) as measured by fluorescence-activated cell sorting (FACS) analysis.

IC-stimulated tumor necrosis factor (TNF)-α and interleukin (IL)-6 production in peripheral blood mononuclear cells: Human peripheral blood mononuclear cells in media containing 10% FBS and various concentrations of BMS-986142 were stimulated for 7 hours at 37°C with ICs prepared from AffiniPure F(ab’)2 fragment goat anti-human and human IgG (Jackson ImmunoResearch), both of which were purified to remove endotoxin prior to IC generation. TNF-α and IL-6 levels were measured using enzyme-linked immunosorbent assay (ELISA; TNF-α OptEIA, BD Biosciences; IL-6 OptEIA, BD Biosciences). For analogous experiments measuring the effect of Toll-like receptor 4 stimulation on these endpoints, lipopolysaccharide (Sigma-Aldrich, St. Louis, MO, USA) was used as the stimulus.

Whole blood assays of BCR-stimulated cluster of differentiation (CD) 69 expression on B cells: To measure BCR-stimulated B cells, anticoagulant citrate dextrose solution A-treated human whole blood was incubated with various concentrations of BMS-986142 and stimulated with 30 μg/mL AffiniPure F(ab’)2 fragment goat anti-human IgM, endotoxin cleared (Jackson ImmunoResearch), and 10 ng/mL human IL-4 (Peprotech, Rocky Hill, NJ, USA) for 18 hours at 37°C with agitation. The cells were stained with fluorescein isothiocyanate (FITC)-conjugated mouse anti-human CD20 (BD Pharmingen, San Diego, CA, USA) and phycoerythrin (PE)-conjugated mouse anti-human CD69 monoclonal antibody (BD Pharmingen), lysed, fixed, and then washed. The amount of CD69 expression was quantitated by MFI after gating on the CD20-positive B-cell population measured by FACS analysis.

Whole blood assays of Fcε receptor I (anti-IgE)-stimulated CD63 expression on basophils: Human whole blood (ethylenediaminetetraacetic acid [EDTA] treated) was diluted fourfold and pre-incubated with various concentrations of BMS-986142 for 30 minutes at 37°C and then stimulated with 0.9 μg/mL goat anti-human IgE endotoxin cleared (Invitrogen, Grand Island, NY, USA) and stained with PE-conjugated mouse anti-human C-C chemokine receptor type 3 and FITC-conjugated mouse anti-human CD63 monoclonal antibodies (American Laboratory Products Company [ALPCO], Salem, NH, USA) for 25 minutes at 37°C. The blood was lysed, fixed, and then washed. The percentage of CD63+ cells was measured after gating on the C-C chemokine receptor type 3-positive basophil population by FACS analysis.

RANK-L-induced osteoclastogenesis: Osteoclast precursor cells (Lonza Group, Basel, Switzerland) were cultured at 37°C in basal medium supplemented with 10% FBS, 25 ng/mL human macrophage colony-stimulating factor, 60 ng/mL human RANK-L, and 2 mM L-glutamine, and containing various concentrations of BMS-986142. After 9 days in culture, cells were fixed and stained for tartrate-resistant acid phosphatase (TRAP; Sigma-Aldrich, St. Louis, MO, USA), and the number of TRAP-positive multinucleated cells (>2 nuclei per cell) was measured as described previously [22].

### *In vivo* studies

All animal procedures were conducted with the approval of the Bristol-Myers Squibb Animal Care and Use Committee and Committee for the Purpose of Control and Supervision of Experiments on Animals (CPCSEA; registration number 1089/RO/bc/2007/CPCSEA) and Government of India guidelines. Mice (Harlan Laboratories, Indianapolis, IN, USA and Netherlands) were housed under a 12-hour/12-hour light/dark cycle and provided standard access to rodent chow diet and fresh drinking water *ad libitum*. Blood was collected at 1, 4, and 24 hours after the morning dose on day 14 for measurement of BMS-986142 levels. Mice were sacrificed by CO_2_ euthanasia.

#### Primary anti-keyhole limpet hemocyanin (KLH) antibody responses in mice

Activity against a KLH-induced antibody response in mice was used as a measure of antigen-driven B-cell responses. Female BALB/c mice (8–12 weeks old) were immunized intraperitoneally (IP) with 250 μg of KLH (Pierce, Rockford, IL, USA) in phosphate-buffered saline on day 0. Mice in appropriate groups were dosed once daily (QD) by oral (PO) gavage with BMS-986142 (3, 10, and 30 mg/kg) in polyethylene glycol (PEG) 400:water (80:20 [v/v]). Blood was collected on days 7 and 14 after immunization, and serum was separated and analyzed for anti-KLH IgM titers (day 7) and anti-KLH IgG titers (day 14) using an enzyme-linked immunosorbent assay (ELISA). Captured anti-KLH antibodies were detected using horseradish peroxidase-conjugated antibody specific for mouse IgM or IgG (Southern Biotechnology Associates, Birmingham, AL, USA) and the 3,3′,5,5′-tetramethylbenzidine peroxidase substrate system (Kirkegaard and Perry Laboratories, Gaithersburg, MD, USA). Optical densities of developed plates were quantitated in a SpectraMax Plus ELISA plate reader (Molecular Devices, Sunnyvale, CA, USA). Sera collected from mice on day 7 or day 14 after immunization with KLH were pooled and used as a standard comparator. Data were expressed in relation to vehicle control titers, which were assigned a value of 1.

#### Collagen antibody-induced arthritis (CAIA) mouse model

A mixture of 4 monoclonal anti-mouse type II collagen antibodies (1 mg of each) was administered IP to female BALB/c mice (Harlan Laboratories; 8–10 weeks old) [23]. PO QD dosing was immediately started with BMS-986142 at 3, 10, and 30 mg/kg in ethanol:tocopherol PEG 1000 succinate:PEG300 (EtOH:TPGS:PEG300; 5:5:90). Three days later, mice were injected IP with 1.25 mg/kg LPS from *Escherichia coli* O111:B4 (Sigma-Aldrich). Mice were monitored daily for development and severity of paw inflammation as described previously [24].

For mouse whole blood, stimulation used AffiniPure F(ab’)2 fragment goat anti-mouse IgG + IgM (Jackson ImmunoResearch) at 200 μg/mL and 1 ng/mL mouse IL-4 (R&D Systems, Minneapolis, MN, USA) for 5 hours at 37°C with agitation. After staining with APC rat anti-mouse CD19 antibody (BD Biosciences) to identify the B cells, the amount of CD69 expression was quantitated by FACS using FITC-conjugated anti-mouse CD69 monoclonal antibody (BD Biosciences).

#### Collagen-induced arthritis (CIA) mouse model

Induction of CIA was performed as described previously [25]. In brief, male DBA/1 mice (Harlan Laboratories, 8–10 weeks old) were injected subcutaneously at the base of the tail with bovine type II collagen (200 μg) admixed with reconstituted Sigma Adjuvant System (Sigma-Aldrich). The mice were boosted 21 days later in the same manner. For preventative administration, PO QD dosing was immediately started with BMS-986142 in EtOH:TPGS:PEG300 (5:5:90); for therapeutic administration, start of dosing was delayed until the booster immunization on day 21. For BMS-986142 plus MTX preventative studies, mice received vehicle; BMS-986142 at 4, 10, or 30 mg/kg; BMS-986142 at 4 mg/kg plus MTX 0.25 mg/kg; or MTX at 0.25 mg/kg daily. For BMS-986142 plus etanercept (Enbrel^®^; Amgen, Thousand Oaks, CA, USA) therapeutic studies, mice received vehicle daily; BMS-986142 at 2, 4, or 25 mg/kg daily; BMS-986142 at 2 or 4 mg/kg daily plus etanercept at 15 mg/kg IP twice weekly (BIW); or etanercept at 15 mg/kg IP BIW. For BMS-986142 plus murine cytotoxic T lymphocyte-associated protein 4 immunoglobulin (CTLA-4-Ig) preventative studies, mice received vehicle daily; BMS-986142 at 10 or 30 mg/kg daily; murine CTLA-4-Ig at 0.05 or 0.2 mg/kg IP BIW; or BMS-986142 at 10 mg/kg daily plus murine CTLA-4-Ig at 0.05 or 0.2 mg/kg IP BIW. Dosing proceeded from day 0 through study completion (36 days).

Following the booster immunization, mice were monitored 3 times per week for development and severity of paw inflammation as described previously [24]. Blood was collected after the morning dose on the last day of the study for measurements of circulating blood levels of BMS-986142 and to determine levels of mouse anti-bovine type II collagen IgG antibodies by ELISA (anti-type II collagen IgG antibody assay kit; Chondrex, Richmond, WA, USA). On the last day of the study, spleens were isolated and processed into single-cell suspensions using a GentleMACS dissociator and ammonium-chloride-potassium lysing buffer (Invitrogen). The percentage of B220lowCD138+ plasma cells was measured by flow cytometry, along with expression of CD38 MFI on plasma cells, after gating the live, single CD3-negative cell population. Bone morphology of CIA mice was evaluated by micro-computed tomography (micro-CT) using the Scanco VivaCT40 (Scanco Medical AG, Zurich, Switzerland). Imaging parameters included approximately 500 slices of 21-μm thickness acquired with 250 projections, 500 ms integration time, 55 kVp of photon energy, and 145 μA of current. Region of interest focused on the hind/mid-foot sections (talus to proximal end of the first metatarsal bone). Threshold settings were optimized using histomorphometric methods. Bone mineral density (BMD) and bone surface area were evaluated using a hydroxylapatite calibration phantom and Scanco proprietary software. Histological evaluation of the right hind paws was conducted, and lesions were scored on a severity scale from 0 (normal) to 4 in two separate categories, inflammation (cellular infiltration, pannus formation, and edema) and bone resorption, as described previously [24].

### Statistical analyses

Half maximal inhibitory concentration (IC50) values were derived using non-linear regression analysis. Data for animal studies were presented as mean ± standard error of the mean. KLH antibody titer data were analyzed using one-way analysis of variance (ANOVA) and Dunnett’s post-test for multiple comparisons. Arthritis clinical score data were analyzed using Kruskal–Wallis with Dunn’s post-test for multiple comparisons. For histological evaluation, scores for each group were compared using one-way ANOVA and Tukey’s multiple comparison test.

## Results

### *In vitro* characteristics

#### BMS-986142 potency against, and selectivity for, BTK

BMS-986142 potently inhibited human recombinant BTK with an IC50 of 0.5 nM in enzymatic assays. Against a panel of 384 kinases, BMS-986142 was highly selective, with only five other kinases inhibited with <100-fold selectivity for BTK as shown in Table 1. Four of these kinases were Tec family kinases, of which BTK is a member, and only Tec (IC50 = 10 nM) was inhibited with <30-fold selectivity compared with BTK.

**Table 1:** Kinase selectivity profile of BMS-986142.

Kinase	Biochemical IC50 (nM)	Fold selectivity for BTK
BTK	0.5	–
Tec	10	20×
ITK	15	30×
BLK	23	46×
Txk	28	56×
BMX	32	64×
Lck	71	142×
FGR	81	162×
CSK	148	296×
IGF1R	247	494×
Src	1115	2047×

Enzymatic IC50 data on kinases showing <20% control at 1000 nM BMS-986142 against a panel of 384 kinases in DiscoveRx (Fremont, CA, USA; formerly Ambit Biosciences) panel.

#### BMS-986142 activity in B cells, peripheral blood mononuclear cells, and whole blood

Because of its essential role in BCR signaling [5, 8, 26], the effect of BTK inhibition by BMS-986142 on B-cell function was evaluated (Table 2). In primary human B cells stimulated through the BCR, BMS-986142 inhibited multiple functional endpoints, including production of inflammatory cytokines (IL-6 and TNF-α), cell proliferation, and surface expression of CD86 on B cells or on memory B cells. The CD40 receptor is not dependent on the catalytic activity of BTK to mediate downstream signaling [27] and, as anticipated, BMS-986142 did not inhibit CD40L-induced expression of CD86 or CD69 on peripheral blood B cells (IC50, >10,000 nM for both). Collectively, these results support the high functional selectivity of BMS-986142 in B cells.

**Table 2:** Potencies against functional endpoints in human B cells and peripheral blood mononuclear cells.

Cell	Receptor (Stimulus)	Endpoint	BMS-986142 (IC50, nM)
B cells (Ramos)	BCR (anti-IgM)	Ca^2+^ flux	9 ± 5
B cells (tonsillar)	BCR (anti-IgM)	Proliferation	3 ± 1
B cells (peripheral blood)	BCR (anti-IgM)	CD86 expression	4 ± 3
Memory B cells (peripheral blood)	BCR (anti-IgM)	CD86 expression	3 ± 3
B cells (tonsillar)	BCR (anti-IgM)	TNF-α	3 ± 3
B cells (tonsillar)	BCR (anti-IgM)	IL-6	5 ± 3
B cells (peripheral blood)	CD40 (CD40L)	CD86 expression	>10,000
B cells (peripheral blood)	CD40 (CD40L)	CD69 expression	>10,000
Peripheral blood mononuclear cells	FcγR (immune complex)	TNF-α	3 ± 3
Peripheral blood mononuclear cells	FcγR (immune complex)	IL-6	4 ± 3
Peripheral blood mononuclear cells	TLR4 (LPS)	TNF-α	>30,000

**Fig 1:**
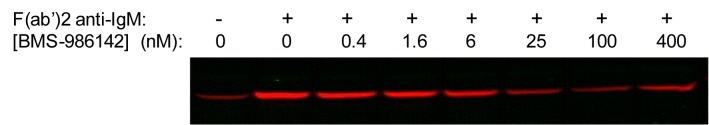
Inhibition of anti-IgM-stimulated phosphorylation of phospholipase C-γ2 in Ramos B cells by BMS-986142.

Upon BCR activation, BTK phosphorylates phospholipase C (PLC)-γ2, which induces intracellular calcium mobilization [19, 28–30]. When Ramos B cells were treated with anti-IgM to activate BCR, BMS-986142 inhibited BTK-dependent calcium flux with an IC50 of 9 nM (Table 2). Furthermore, Ramos B cells exhibited concentration-dependent inhibition of BTK-catalyzed phosphorylation of PLCγ2 with a similar potency (IC50 of approximately 20 nM) as shown in Fig 1.

Previous studies of BTK inhibition indicate that BTK is essential in FcγR-mediated inflammatory cytokine production [5]. Therefore, we investigated the effect of BMS-986142 on the ability of peripheral blood mononuclear cells to produce cytokines. BMS-986142 inhibited TNF-α and IL-6 production (IC50, 3 and 4 nM, respectively; Table 2) in these cells stimulated through IgG-containing IC-driven, low affinity-activating FcγR (FcγRIIa and FcγRIIIa). Potencies were equivalent to those measured against BCR-dependent functional endpoints in B cells. LPS stimulation of TNF-α in peripheral blood mononuclear cells was not inhibited by BMS-986142 (IC50, >30,000 nM), consistent with published reports showing TLR4 signaling is not dependent on BTK catalytic activity [27].

In assays using human whole blood, BMS-986142 inhibited BCR-stimulated expression of CD69 on B cells (IC50 = 90 nM). A similar potency was obtained against FcεRI-driven CD63 surface expression on basophils in human blood (IC50 = 89 nM), consistent with the reported role of BTK in mediating signal transduction through this receptor in granulocytes [31].

#### Inhibition of RANK-L-dependent osteoclastogenesis

**Fig 2:**
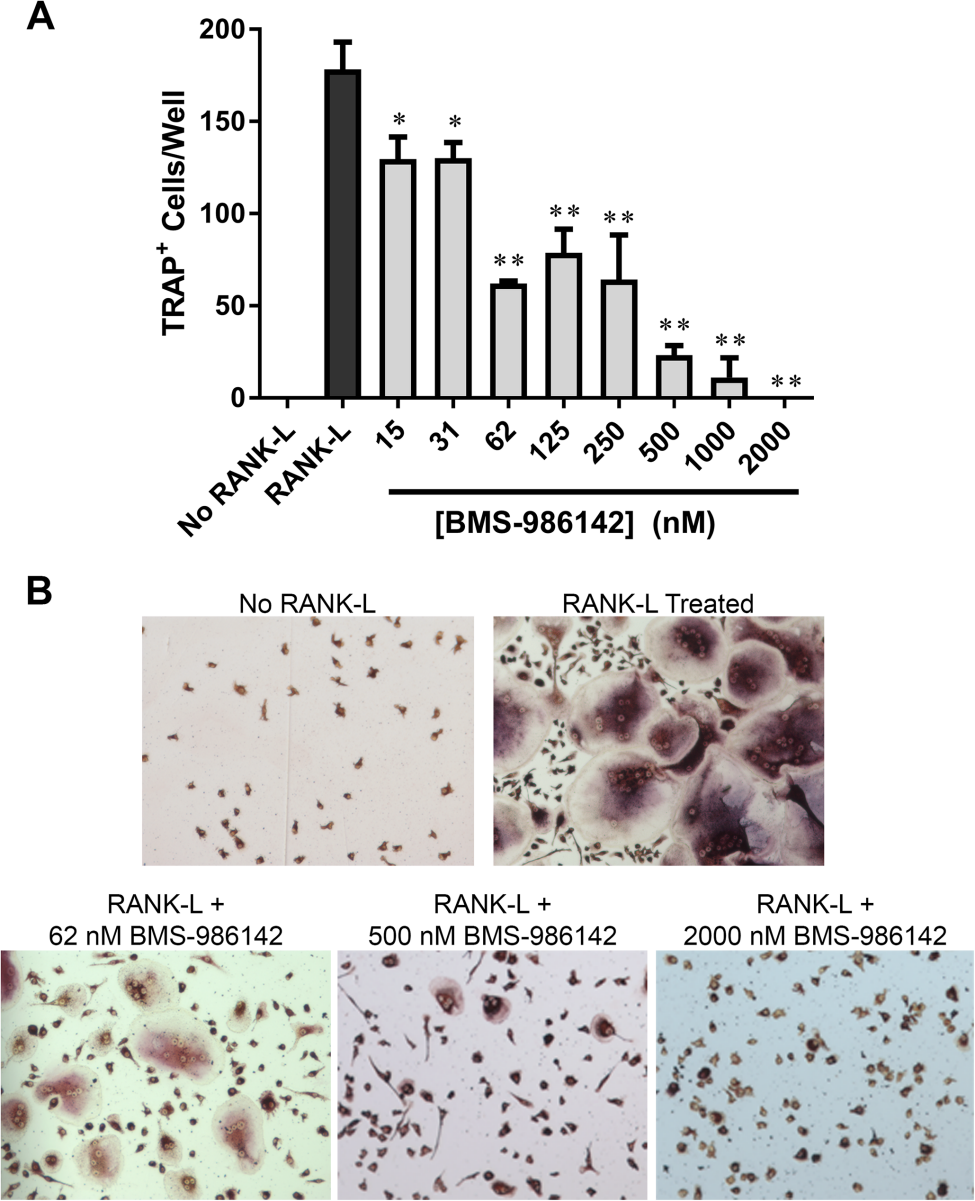
BMS-986142 inhibits RANK-L-induced osteoclastogenesis in human monocytic precursors. (**A**) Quantitation of the number of TRAP-positive multinucleated cells per well after 9 days in culture. Data shown are mean ± standard deviation. ^*^p < 0.05, ^**^p < 0.01 versus vehicle group, n = 3/condition. (**B**) Representative images.

Because BTK regulates RANK-dependent osteoclastogenesis [13, 14], which drives bone resorption in RA [32], we explored the effect of BMS-986142 on this function. Stimulation of primary monocytic progenitor cells with RANK-L resulted in large, multinucleated osteoclasts that stained positively for TRAP (Fig 2). BMS-986142 dose-dependently inhibited this RANK-L-dependent formation of osteoclasts (Fig 2A). Inhibition was evident at BMS-986142 concentrations as low as 15 nM, showing that BMS-986142 inhibited RANK-L-induced osteoclastogenesis with a potency consistent with other BTK-dependent cellular assays. At higher concentrations of BMS-986142, the cells resembled mononuclear cells not exposed to RANK-L (Fig 2B). These results suggest that BMS-986142 may impact bone resorption in RA directly through effects on osteoclastogenesis, although it is important to note that the matrix degradation potential of these multinucleated TRAP-positive cells was not assessed.

### *In vivo* pharmacology

#### Primary anti-KLH antibody responses

**Fig 3:**
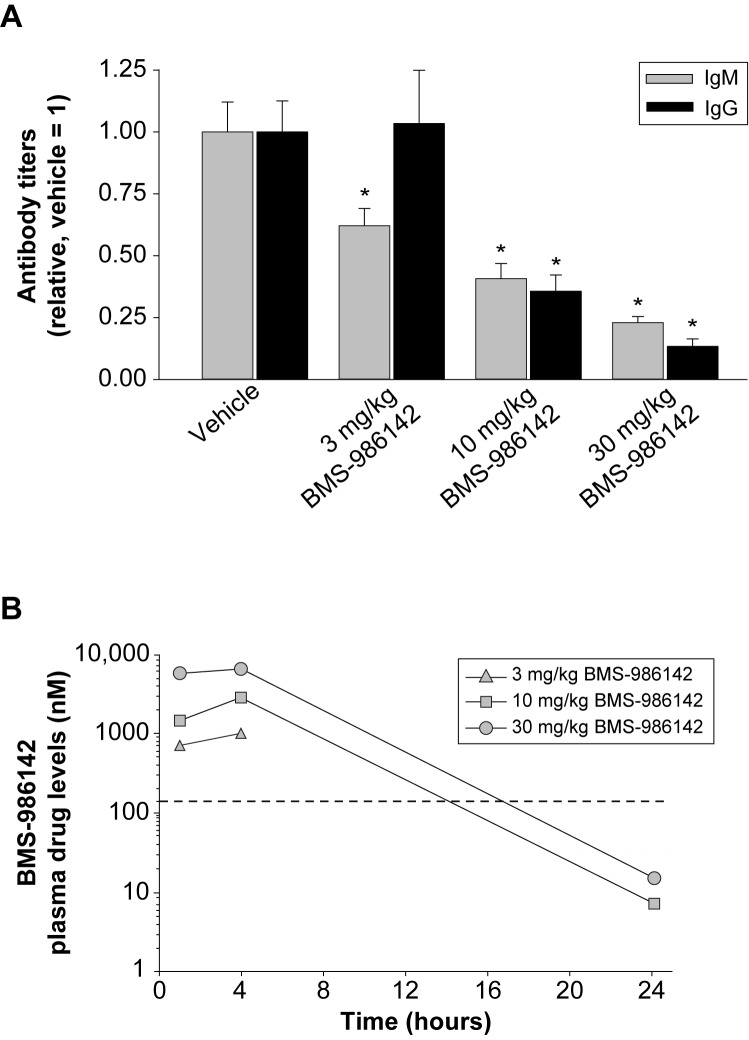
BMS-986142 blocks neoantigen-induced antibody responses. (**A**) Primary anti-KLH antibody response over 14 days in mice: day 7 IgM (gray bars) and day 14 IgG (black bars) anti-KLH titers. Data shown are mean ± SEM. (**B**) Pharmacokinetics of BMS-986142 measured on day 14 of the study with the data represented as time after the morning dose. The dashed line represents the IC50 value determined *in vitro* against BCR-stimulated CD69 expression on B cells in mouse whole blood. ^*^p < 0.05 versus vehicle group, *n* = 7–10/group.

As a measure of antigen-induced B-cell responses *in vivo*, BMS-986142 was evaluated for its effect on anti-KLH antibody responses in mice immunized with KLH. As shown in Fig 3A, BMS-986142 dose dependently inhibited anti-KLH IgM and IgG antibody responses at days 7 and 14, respectively. Significant reductions occurred at doses of 10 and 30 mg/kg, with the high dose providing 77% and 87% inhibition of IgM and IgG responses, respectively. On day 14 of the study, BMS-986142 concentrations were measured and showed that the drug levels were maintained above the mouse whole blood BCR-stimulated CD69 IC50 of 140 nM for <11, 14, and 17 hours at doses of 3, 10, and 30 mg/kg, respectively (Fig 3B). Note that drug levels at the 24 hour time point at the low dose of 3 mg/kg were below the limit of detection.

#### BMS-986142 activity in the CAIA mouse model

**Fig 4:**
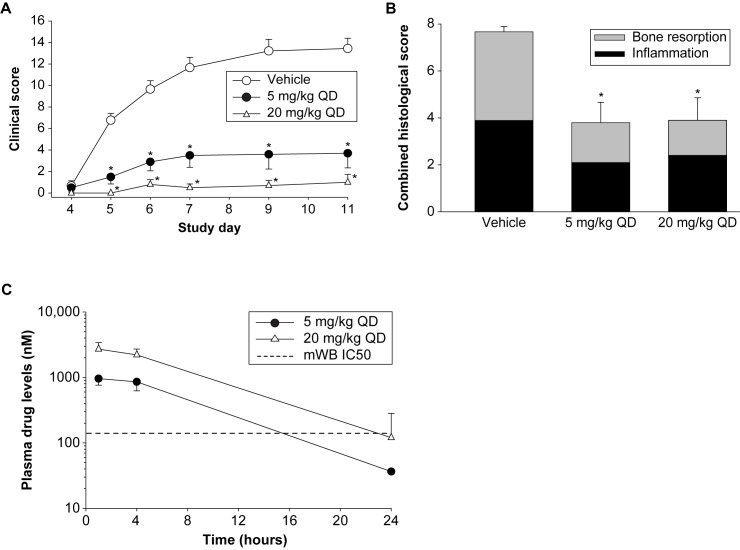
BMS-986142 is efficacious in the murine CAIA model. (**A**) Mean clinical scores, (**B**) histological evaluation of the right hind paws of CAIA mice, and (**C**) pharmacokinetics of BMS-986142 on day 12 of the CAIA study. Data shown are mean ± SEM. ^*^p < 0.05 versus vehicle group, n = 7–10/group.

To investigate the effect of BTK inhibition on FcγR-dependent functions important in the pathobiology of inflammatory arthritis, BMS-986142 was evaluated in the CAIA mouse model. While the CAIA model is dependent on FcγR-driven mechanisms [33], it is not dependent on either B or T cells, as lymphocyte-deficient mice are susceptible to CAIA [34]. Prophylactic administration of BMS-986142 reduced the incidence and severity of clinically evident disease. Clinical scores at study end were reduced by 72% with BMS-986142 at 5 mg/kg, and 20 mg/kg BMS-986142 provided essentially complete protection from disease (>90% inhibition; Fig 4A).

In order to determine whether BMS-986142 has a protective effect on bone and cartilage, tibiotarsal joints of the hind paws were histologically evaluated for severity of inflammation, synovial hyperplasia, bone resorption, and cartilage erosion. Doses of 5 and 20 mg/kg were effective at reducing bone resorption as well as inflammation scores (Fig 4B). BMS-986142 at 5 mg/kg provided approximately 16 hours of daily coverage of the mouse whole blood BCR-stimulated CD69 IC50 of 140 nM (Fig 4C). Near-complete (24-hour) coverage was obtained with the 20 mg/kg dose.

#### BMS-986142 activity in the CIA mouse model

**Fig 5:**
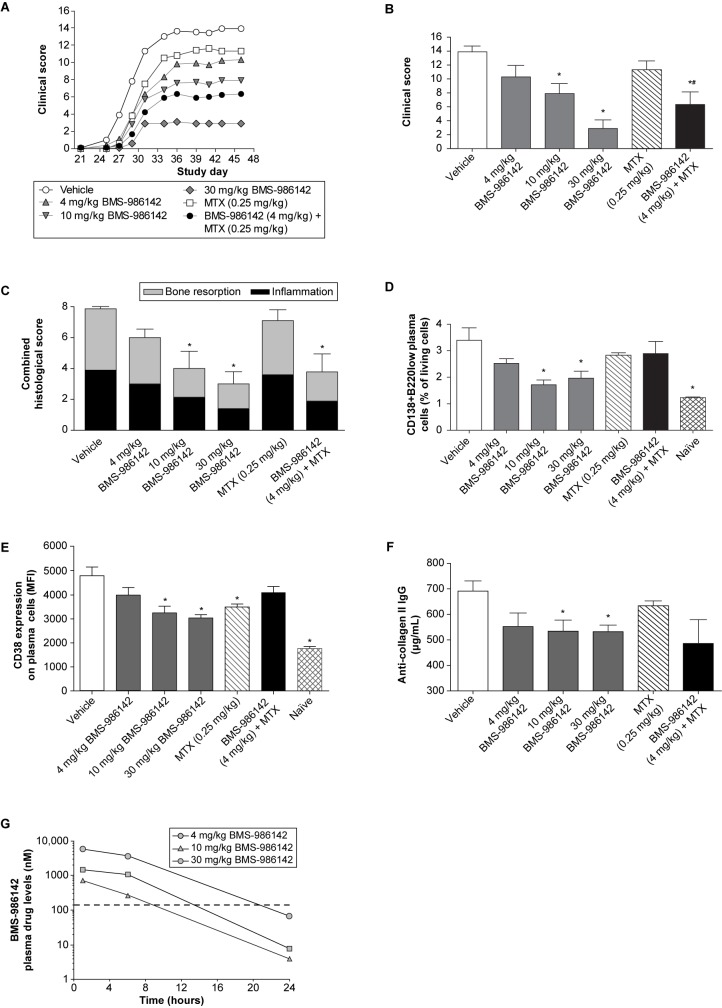
BMS-986142 is efficacious against CIA in mice. (**A**) Mean clinical scores over the course of the study, (**B**) mean clinical scores at the end of the study (day 46), (**C**) histological evaluation of the right hind paws, (D) plasma cells as measured by FACS analysis performed on spleens from 5 mice per group (3 mice in naïve group; non-immunized mice), (E) CD38 expression (MFI) on splenic CD138+B220low plasma cells, (F) anti-collagen II IgG titers, and (**G**) pharmacokinetics of BMS-986142 measured on the last day of the study with the data represented as time after the morning dose. The dashed line represents the IC50 value determined *in vitro* against BCR-stimulated CD69 expression on B cells in mouse whole blood. Data for **B** through **F** shown as mean ± SEM. ^*^p < 0.05 versus vehicle group, ^#^*p* < 0.05 versus either treatment alone, n = 10/group.

The CIA mouse model exhibits similarities in many of the underlying pathobiologic mechanisms of RA and demonstrates a central role for T and B cells [35–37]. When given according to a preventative administration schedule, BMS-986142 at 4, 10, and 30 mg/kg resulted in dose-dependent reductions of 26%, 43%, and 79% in clinically evident disease, respectively, at the end of the study (Fig 5A and 5B). Interestingly, 4 mg/kg BMS-986142 provided an additive benefit in clinical scores (54% inhibition) when co-administered with MTX versus 19% inhibition with MTX alone, indicating that BMS-986142 is not only effective as monotherapy but provides additive benefits when co-administered with MTX.

Histological evaluation of the joints showed that protection from inflammation and bone resorption was dose dependent and mirrored effects on clinical scores. Consistent with the effects of combination treatment observed against clinically evident disease, co-administration of BMS-986142 at 4 mg/kg with MTX resulted in a 53% reduction in inflammation and bone resorption compared with 24% and 10%, respectively, with either drug alone (Fig 5C). While treatment with BMS-986142 did not deplete B cells, the disease-associated increases in splenic CD138+B220low plasma cells (Fig 5D) and expression of CD38 on plasma cells normally induced by disease (Fig 5E) were inhibited, showing that BTK inhibition can maintain the B-cell repertoire toward a disease-free state. Furthermore, serum anti-collagen II IgG titers (Fig 5F) were significantly inhibited with 10 and 30 mg/kg BMS-986142. Pharmacokinetic measurements showed that doses at 4, 10, and 30 mg/kg provided 9, 14, and 21 hours of daily coverage of the mouse whole blood BCR-stimulated CD69 IC50 of 140 nM, respectively (Fig 5G).

**Fig 6:**
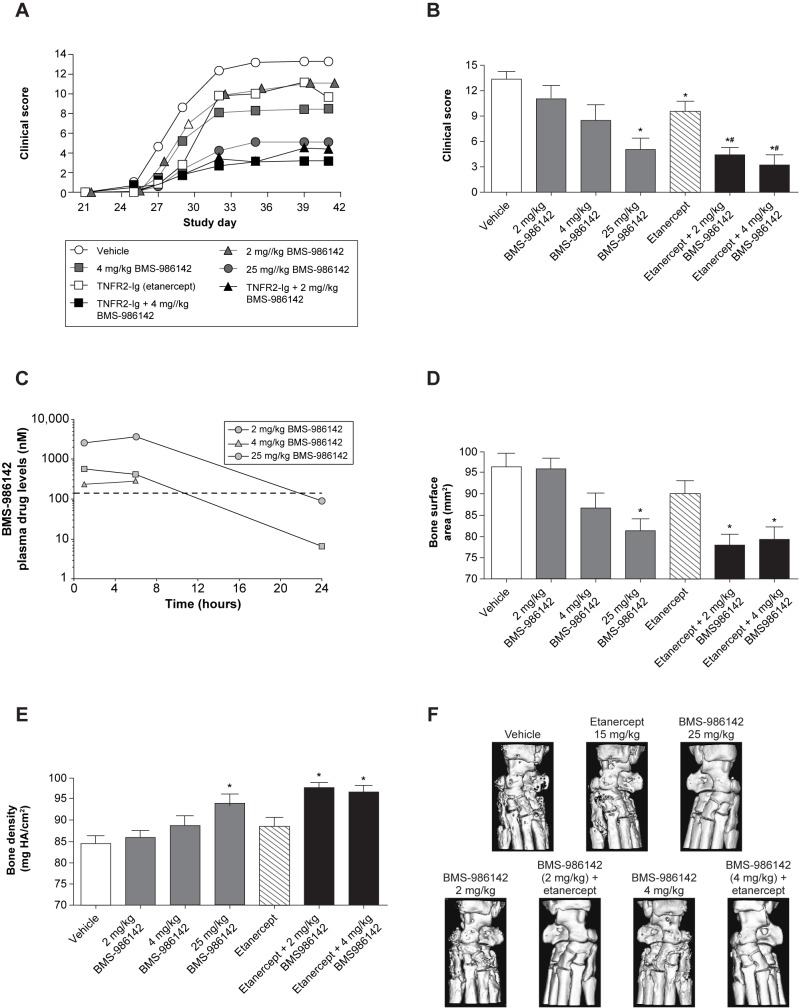
Therapeutic treatment with BMS-986142 co-administered with etanercept protected from CIA in mice. (**A**) Mean clinical scores over the course of the study, (**B**) mean clinical scores at the end of the study (day 41), (**C**) pharmacokinetics of BMS-986142 measured on the last day of the study with the data represented as time after the morning dose, (**D**) bone surface area measurements by micro-CT of the hind limbs, (**E**) bone mineral density measurements by micro-CT of the hind limbs, and (**F**) representative images of treatment groups using micro-CT. The dashed line represents the IC50 value determined *in vitro* against BCR-stimulated CD69 expression on B cells in mouse whole blood. Data for B, D, and E shown as mean ± SEM. ^*^p < 0.05 versus vehicle group, ^#^*p* < 0.05 versus either treatment alone, n = 9–10/group.

BMS-986142 also produced dose-dependent reductions in clinical scores when administration was delayed until the collagen booster on day 21 (Fig 6A and 6B). BMS-986142 doses of 2, 4, and 25 mg/kg in this therapeutic dosing regimen resulted in clinical score reductions of 17%, 37%, and 67%, respectively, at the end of the study. Consistent with the reduction in clinical scores, BMS-986142 provided dose-dependent protection against disease-induced remodeling/erosions and loss of bone mass compared with vehicle. Pharmacokinetic measurements showed that doses of 2, 4, and 25 mg/kg provided <10, 11, and 22 hours of daily coverage of the mouse whole blood BCR-stimulated CD69 IC50 of 140 nM, respectively (Fig 6C).

#### BMS-986142 co-administered with etanercept or CTLA-4-Ig in the CIA mouse model

In addition to treating with BMS-986142 as monotherapy, the benefits of combining BMS-986142 with etanercept was also investigated. Compared with either treatment alone, co-administration of low-dose (2 mg/kg) or mid-dose (4 mg/kg) BMS-986142 plus etanercept resulted in significant benefit, as shown in Fig 6B. Micro-CT scans of the hind paw showed that BMS-986142 provided dose-dependent protection against disease-induced remodeling/erosions and loss of bone mass evident in the mice receiving only vehicle, mirroring effects on clinical scores. Significant effects on bone surface area and BMD were also observed (Fig 6D–6F).

**Fig 7:**
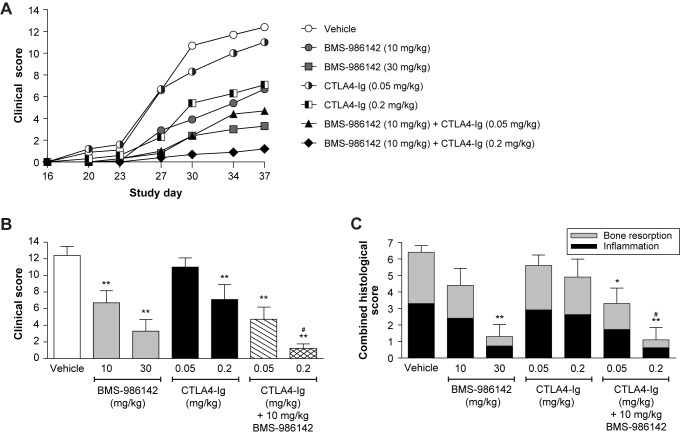
BMS-986142 co-administered with CTLA-4-Ig shows an enhanced effect against CIA in mice. (**A**) Mean clinical scores over the course of the study, (**B**) mean clinical scores at the end of the study (day 37); BMS-986142 was administered by oral gavage once daily and CTLA-4-Ig by intraperitoneal injection twice weekly, and (**C**) total inflammation and bone resorption histology scores of the hind paws. Data shown as mean ± SEM. ^*^p < 0.05 versus vehicle group, ^**^p < 0.01 versus vehicle group, ^#^*p* < 0.05 versus either treatment alone.

Co-administration of a suboptimal dose of BMS-986142 (10 mg/kg PO QD) with murine CTLA-4-Ig also provided an additive benefit (Fig 7A and 7B). Clinical scores were reduced by 46% with BMS-986142 at 10 mg/kg, by 11% and 43% with CTLA-4-Ig at 0.05 and 0.2 mg/kg, respectively, and by 62% and 90% with the respective combinations. BMS-986142 at 10 mg/kg plus CTLA-4-Ig at 0.2 mg/kg also provided protection from inflammation and bone resorption, resulting in 83% inhibition, which mirrored the effects on clinical scores (Fig 7C).

## Discussion

BTK is a key intracellular enzyme that regulates critical pathways involved in the pathobiology of RA and other autoimmune disorders. In the studies reported herein, BMS-986142 was found to be a potent and selective inhibitor of BTK that effectively blocked BCR-dependent functional endpoints in human B cells, FcγR-dependent cytokine production from peripheral blood mononuclear cells, and RANK-L-induced osteoclastogenesis.

B cells play multiple roles in RA pathobiology, including production of autoantibodies and inflammatory cytokines, and as antigen-presenting cells [4]. Therefore, inhibition of antigen-driven B-cell functions by BMS-986142 may attenuate these effects in RA. Inhibition of RANK-L-induced osteoclastogenesis further supports BMS-986142 as an attractive therapeutic option for RA, where bone loss results from increased osteoclastic bone resorption [38]. IgG-containing ICs trigger FcγR-mediated TNF-α secretion and are critical in the development of RA [33, 39, 40]. BTK inhibition of IgG-containing, IC-driven, FcγR-dependent functions in myeloid cells by BMS-986142 represents an important potential mechanism for the suppression of RA pathobiology and is consistent with previous studies of BTK inhibitors [5].

Of note, BMS-986142 demonstrated efficacy both as a monotherapy and as a combination therapy in murine models of RA, protecting against clinically evident disease, histologic joint damage, and BMD loss. Importantly, in CIA and CAIA models, efficacy was observed without continuous complete inhibition of BTK (i.e. drug levels providing 14 hours of coverage of mouse whole blood IC50 value), showing effects on both B-cell and FcγR-dependent disease. These results underscore the robust efficacy achieved with BMS-986142 at doses that did not result in continuous complete inhibition of BTK and suggest that combination therapy with a reversible BTK inhibitor may be one route to reducing the potential for significant adverse events during clinical development. Although combining two complementary immunosuppressive mechanisms may carry increased risk of serious infection, a reversible BTK inhibitor such as BMS-986142 may mitigate this risk as precise titration to ensure optimal efficacy could avoid unnecessary and prolonged immunosuppression.

Combining agents that target different pathways, potentially resulting in additive or even synergistic effects, has been shown previously to be beneficial in RA [41]. For example, combination of MTX with biologics and oral small-molecule therapeutics provides superior efficacy with better ACR response and remission rates than either treatment alone [42–44]. In the present work using preclinical models of RA, additive effects were evident when suboptimal doses of BMS-986142 were co-administered with either etanercept, a TNF-α inhibitor, or the murine form of abatacept (CTLA-4-Ig), a T-cell co-stimulation blocker. When BMS-986142 at doses of 2–10 mg/kg were used with standard-of-care treatments, greater improvements in outcomes (i.e. protection from clinically evident disease, bone resorption and inflammation of the paws, and expression of pro-inflammatory cytokines and mediators of autoimmunity) were observed compared with either agent used at equivalent doses alone. This may translate to RA, in the example of anti-TNF combination treatments, as patients with a “lymphoid” phenotype (synovium dominated by B cells and plasmablasts) and with high baseline serum levels of the B-cell chemoattractant CXCL13 showed greater resistance to anti-TNF treatment than patients with RA with a “myeloid” phenotype (synovium rich in inflammatory macrophages) [43]. Therefore, given the importance of B cells in RA pathogenesis, RA patients with a lymphoid synovial phenotype may be particularly well served by treatment with a BTK inhibitor in combination with an anti-TNF biologic. Patients with RA with a myeloid synovial phenotype may also be sensitive to BTK inhibition through FcγR- and RANK-dependent mechanisms. Combining BMS-986142 with abatacept (CTLA-4-Ig) in RA also represents an intriguing possibility based on the present results, especially as these agents represent two complementary mechanisms of action (T-cell co-stimulation and BCR-/FcγR-mediated effects) and would reasonably be expected to lead to enhanced efficacy in combination.

Although the results of the present studies are promising, it is important to acknowledge the limitations of these preclinical models. While CIA in mice is considered to be the preclinical model most reflective of the underlying mechanisms in RA, it is characteristically monogenic with respect to disease course and severity and does not entirely reflect the pathobiology and heterogeneity of RA in humans, thus impacting the ability to determine optimal combination treatments for RA. Moreover, disease in mice can be treated effectively with high monotherapy doses; therefore, suboptimal doses of BMS-986142 and agents representing standard of care were required to demonstrate efficacy of co-administration. Consequently, the relevance of the *in vivo* results to human disease, wherein the lack of complete efficacy upon treatment may reflect differing levels of contributions of these underlying pathobiologic mechanisms across patients rather than suboptimal doses, is not properly recapitulated using the CIA mouse model.

Despite some limitations of these preclinical models, the present results demonstrate that the effects of BTK inhibition with BMS-986142 on antigen-specific BCR-mediated B-cell functions, IgG-containing IC signaling through FcγR in monocytic cells, and RANK-dependent osteoclastogenesis are anticipated to provide clinical benefit in the treatment of autoimmune disorders such as RA. Importantly, these therapeutic advantages are expected to be achievable even without complete and continuous BTK inhibition (as occurs with irreversible BTK inhibitors), making a highly selective, reversible BTK inhibitor such as BMS-986142 an especially intriguing agent to evaluate in the treatment of RA and other autoimmune diseases.

Collectively, the results presented here provide compelling evidence for continuing investigation of BMS-986142 as a treatment option for RA, as a single agent or when co-administered with other agents with complementary mechanisms of action. The safety and efficacy of BMS-986142 are being evaluated in clinical trials in both RA and primary Sjögren’s syndrome, and results will be presented in subsequent reports.

## Abbreviations

CTLA-4: cytotoxic T-lymphocyte-associated protein 4
